# A novel role for cyclooxygenase-2 in regulating vascular channel formation by human breast cancer cells

**DOI:** 10.1186/bcr1626

**Published:** 2006-12-11

**Authors:** Gargi D Basu, Winnie S Liang, Dietrich A Stephan, Lee T Wegener, Christopher R Conley, Barbara A Pockaj, Pinku Mukherjee

**Affiliations:** 1 Department of Biochemistry and Molecular Biology, Mayo Clinic, 13400 E. Shea Blvd., Scottsdale, Arizona 85259, USA; 2 Neurogenomics Division, The Translational Genomics Research Institute, 445 N. Fifth Street, Phoenix, Arizona 85004, USA; 3 Department of Pathology, Mayo Clinic, 13400 E. Shea Blvd., Scottsdale, Arizona 85259, USA; 4 Department of Surgery, Mayo Clinic, 13400 E. Shea Blvd., Scottsdale, Arizona 85259, USA

## Abstract

**Introduction:**

Cyclo-oxygenase (COX)-2 expression correlates directly with highly aggressive and metastatic breast cancer, but the mechanism underlying this correlation remains obscure. We hypothesized that invasive human breast cancer cells that over-express COX-2 have the unique ability to differentiate into extracellular-matrix-rich vascular channels, also known as vasculogenic mimicry. Vascular channels have been associated with angiogenesis without involvement of endothelial cells, and may serve as another mechanism by which tumor cells obtain nutrients to survive, especially in less vascularized regions of the tumor.

**Methods:**

To determine whether COX-2 regulates vascular channel formation, we assessed whether treatment with celecoxib (a selective COX-2 inhibitor) or silencing COX-2 synthesis by siRNA inhibits vascular channel formation by breast cancer cell lines. Cell lines were selected based on their invasive potential and COX-2 expression. Additionally, gene expression analysis was performed to identify candidate genes involved in COX-2-induced vascular channel formation. Finally, vascular channels were analyzed in surgically resected human breast cancer specimens that expressed varying levels of COX-2.

**Results:**

We found that invasive human breast cancer cells that over-express COX-2 develop vascular channels when plated on three-dimensional matigel cultures, whereas non-invasive cell lines that express low levels of COX-2 did not develop such channels. Similarly, we identified vascular channels in high-grade invasive ductal carcinoma of the breast over-expressing COX-2, but not in low-grade breast tumors. Vascular channel formation was significantly suppressed when cells were treated with celecoxib or COX-2 siRNA. Inhibition of channel formation was abrogated by addition of exogenous prostaglandin E_2_. *In vitro *results were corroborated *in vivo *in tumor-bearing mice treated with celecoxib. Using gene expression profiling, we identified several genes in the angiogenic and survival pathways that are engaged in vascular channel formation.

**Conclusion:**

Antivascular therapies targeting tumor cell vasculogenic mimicry may be an effective approach to the treatment of patients with highly metastatic breast cancer.

## Introduction

Tumor growth and metastasis are thought to be angiogenesis-related processes [1]. However, it has recently been reported that an angiogenesis-independent pathway, in which tumors can feed themselves without the use of classical blood vessels, exists in very aggressive tumors of the lung and breast, as well as in melanomas [2-4]. This is known as vasculogenic mimicry (VM), a phenomenon in which epithelial tumor cells form vascular channel-like structures to obtain nutrients without the participation of endothelial cells. These laminin-rich channel-like spaces are lined by tumor cells and contain erythrocytes and plasma. These channels are thought to provide a mechanism of perfusion and a dissemination route within the tumor that functions either independently of or simultaneously with angiogenesis [5]. A connection has been suggested between VM and angiogenesis based on the existence of blood flow in the vascular channels [6]. Thus, VM might be an important factor to consider in the design of antivascular therapies. Importantly, it has been shown that breast cancer patients who exhibit VM in their resected tumors have a lower 5-year survival than do patients without VM [7].

Although a correlation has been found between the presence of VM and poor clinical outcome, little is known regarding the molecular composition and regulation of these channels. Based on microarray analysis of melanoma cells, the biologically relevant proteins in VM were vascular endothelial cadherin (VE-cadherin), erythropoietin-producing hepatocellular carcicnoma-A2 (EPHA2), matrix metalloproteinases (MMPs), and laminin 5-γ-2-chain (LAMC2). Independently reducing their levels of expression resulted in complete inability of aggressive melanoma cells to execute VM in three-dimensional culture conditions [8,9]. Furthermore, xenografts generated from inflammatory breast cancer (IBC) cells that execute VM *in vitro *expressed higher levels of angiogenic factors than did xenografts from non-IBC cells that did not execute VM. These angiogenic factors included angiogenin-1, vascular endothelial growth factor (VEGF), basic fibroblast growth factor (bFGF), flt-1, integrin-β_3_, and CD31. It has been suggested that upregulation of angiogenesis-related genes may result in the observed vascular phenotype of IBC tumor cells [10]. Therefore, it is important to investigate whether known antiangiogenic agents might prevent VM. A recent report [11] suggested that specific antiangiogenic agents such as anginex, TNP-470, and endostatin had minimal effect on VM in human melanoma MUM-2B and C8161 cells, suggesting differential response of endothelial cell dependent angiogenesis and VM. It is therefore of great importance to investigate additional factors that may regulate vascular channel formation and determine whether inhibiting those factors might prevent VM.

Over-expression of cyclo-oxygenase (COX)-2 is known to correlate with the aggressive and invasive potential of tumor cells by several mechanisms [12]. One of the mechanisms modulated by COX-2 during carcinogenesis is angiogenesis, presumably through increased production of proangiogenic factors such as VEGF and IL-8 [13]. Similarly, COX-2-specific inhibitors reduce angiogenesis by inhibiting mitogen activated protein kinase (extracellular signal regulated kinase [ERK]2) activity and by interfering with nuclear translocation of ERK [14]. To determine whether COX-2 regulates vascular channel formation, we assessed whether treatment with celecoxib (a selective COX-2 inhibitor) or silencing COX-2 synthesis by siRNA inhibits vascular channel formation. The aims of our study were to compare the ability of human breast cancer cells expressing high and low levels of COX-2 to form vascular channels on three-dimensional matrigel cultures, and to assess the effect of therapeutically targeting COX-2 *in vitro *and *in vivo *on VM. In additional, we sought to identify candidate genes that are involved in COX-2-induced channel formation by microarray analysis and, finally, to correlate the cell line data with findings in surgically resected human breast cancer specimens.

## Materials and methods

### Cell culture

The human breast cancer cell lines MDA-MB-231, MDA-MB-435, MCF-7, and ZR-75-1 were obtained from the American Type Culture Collection (Rockville, MD, USA). Briefly, cells were grown in Dulbecco's modified Eagle medium (DMEM) supplemented with 5% fetal calf serum, 100 U penicillin, 0.1 μg streptomycin, and 2 mmol/l L-glutamine. Cells were maintained at log phase at 37°C with 5% carbon dioxide.

### Assay for vasculogenic mimicry

The assay was performed as described previously [14]. Briefly, cells were grown until they were about 80% confluent. Cells (4 × 10^4 ^cells/ml) were plated with increasing concentrations of celecoxib (a specific COX-2 inhibitor; from 40 to 60 μmol/l). Dimethyl sulfoxide (DMSO; vehicle) was used as a negative control. In some experiments, exogenous prostaglandin (PG)E_2 _(50 ng/ml) was added to the cells in addition to celecoxib (40 μmol/l, which is the 50% inhibitory concentration for MDA-MB-231 cells). Celecoxib concentrations were based on our previously reported study [15]. Similar doses were also utilized by other investigators on additional cell lines [16-18]. The optimal dose of exogenous PGE_2 _was determined by titrating doses (25, 50, 100, 200, 400, 800, 1600, 3200, and 6400 ng/ml) of PGE_2 _with 40 μmol/l celecoxib (data not shown). The optimal dose of 50 ng/ml PGE_2 _was based on low cell death accompanied with reversal of celecoxib-induced inhibition of channel formation. Beyond 200 ng/ml PGE_2_, significant cell death was observed.

A 24-well tissue culture plate was evenly coated with 0.1 ml/well growth factor-reduced matrigel (BD Biosciences, San Jose, CA, USA), which was allowed to solidify at 37°C for 30 min, before the celecoxib-treated cells were plated. The cell suspension was added (1 ml/well) onto the surface of the matrigel and incubated at 37°C for varying times. Cells were photographed using a phase contrast microscope (Nikon USA, Garden City, NY, USA). Vascular channels were quantified by counting the number of connected cells in five randomly selected fields, using × 200 magnification, and dividing the number by the total number of single cells in the same field.

### Small interfering RNA transfections

MDA-MB-231 (high COX-2 expression) cells were plated at 225,000 cells/well in six-well plates and grown to 50% confluence. Cells were transfected with COX-2-specific or control siRNA purchased from Dharmacon (Lafayette, CO, USA) in lipofectamine-2000 transfection reagent (Invitrogen, Carlsbad, CA, USA), in accordance with the manufacturer's instructions. Briefly, for each well, 10 μl transfection reagent was incubated with 500 μl OPTI-MEM medium (Gibco, Carlsbad, CA, USA) for 5 min. Subsequently, the respective siRNA in OPTI-MEM medium was added to the transfection media, resulting in siRNA concentrations of 10, 50, and 100 nmol/l, and added to the wells. Two days after transfection, COX-2 protein expression was determined. siRNA-treated cells were plated on growth factor-reduced matrigel and channel formation was evaluated 48 hours after transfection.

### Protein analysis

Protein lysates were prepared from cell lines in lysis buffer containing 20 mmol/l Hepes, 0.15 M NaCl, and 1% Triton X-100 supplemented with 80 μl/ml phosphatase inhibitor cocktail II (Sigma P-5726, St. Louis, MO, USA) and 10 μl/ml complete protease inhibitor cocktail (Boehringer Mannheim GmbH, Indianapolis, IN, USA). Protein was quantified using the BIO-RAD Protein Assay (BioRad, Hercules, CA, USA) kit with bovine serum albumin as control. Protein (100 μg) was separated by SDS-PAGE (8% gel). Membranes were probed for COX-2 and β-actin. COX-2 antibody was purchased from Cayman Chemical (Ann Arbor, MI, USA) and used at 1:250 dilution. β-Actin antibody was purchased from Santa Cruz Biochemicals (Santa Cruz, CA, USA) and used at 1:200 dilution. Experiments were performed in triplicate and representative Western blots are shown.

### Transmission electron microscopy

Cells were plated on matrigel and fixed in 4% buffered glutaraldehyde and then postfixed in 1% osmium tetroxide. After dehydration in ethanol and propylene, cells were embedded in Epon epoxy resin (Resolution Performance Products, Houston, Texas, USA). Cells were microdissected under magnification to identify areas that exhibited a lobular organization suggestive of the presence of channels. Ultrathin sections stained with uranyl acetate and lead citrate were examined with a Philips EM410 transmission electron microscope.

### Measurement of angiogenic proteins using human angiogenesis array kit

Human Angiogenesis Array I (Ray Biotech Inc., Norcross, GA, USA) consisted of antibodies to 20 proteins spotted in duplicate onto a membrane. The manufacturer's recommended protocol was followed.

### Microarray analysis

Expression profiling was performed to detect alterations in gene expression in the MDA-MB-231 cells treated with 40 μmol/l celecoxib or vehicle for 24 hours by a method previously described [19,20]. Briefly, RNA was extracted using the Qiagen RNAeasy Kit from cells plated on matrigel, and 6 μg cytoplasmic RNA from each sample was converted to double-stranded cDNA using the Superscript Choice System kit and T7-(dT)_24 _primer (100 pmol/μl; Invitrogen, Carlsbad, CA, USA). Report files generated from the emitted fluorescence were reviewed to ensure all quality control standards were met, including percentage of present calls, presence of spike controls, signal scaling factors per chip, and the GAPDH 3'/5' ratios. Expression differences between the experimental and control assays were determined in GeneChip Operating Software (GCOS) from Affymetrix (Santa Clara, CA, USA) and stringent filters on the Detection and Change calls for each probe set were enforced to find those genes with at least a twofold increased or decreased change in expression with the control assay as the baseline.

### Xenografts

Five male athymic nude mice, aged 6 to 8 weeks (NxGen Biosciences Inc., San Diego, CA, USA), were prophylactically treated with either celecoxib (25 mg/kg) or vehicle (DMSO) for 7 days before the tumor cells were inoculated. MDA-MB-231 cells (5 × 10^6^) were suspended in 150 μl serum-free DMEM with an equal volume of cold liquid growth factor reduced matrigel (10 mg/ml) and injected subcutaneously in the mice. All xenografts were excised, fixed in formalin, and paraffin-embedded blocks were sectioned at 7 μm thickness. Histologic evaluation of vascularity was determined by factor VIII staining. Immunohistochemical localization of factor VIII related antigen on endothelial cells was determined using the polyclonal rabbit anti-human Von Willebrand Factor (Dako Cytomation, Carpinteria, CA, USA), using the manufacturers' recommended staining protocol. Red blood cells (RBCs) were detected using Biebrich Scarlet staining. All human breast cancer tissues were obtained from the Mayo Clinic Scottsdale (Department of Pathology) and stained with anti-CD34 (Dako) to identify endothelial cells and Biebrich Scarlet to identify RBCs. COX-2 staining was achieved by using specific goat anti-human COX-2 antibody (Santa Cruz). Grading of the tumors was done according to the Nottingham and modified Bloom Richardson grading criteria. The Mayo Clinic institutional review board approved this research study.

## Results

### Biomechanical potential of invasive breast cancer cells differs from that of non-invasive breast cancer cells

**Figure 1:**
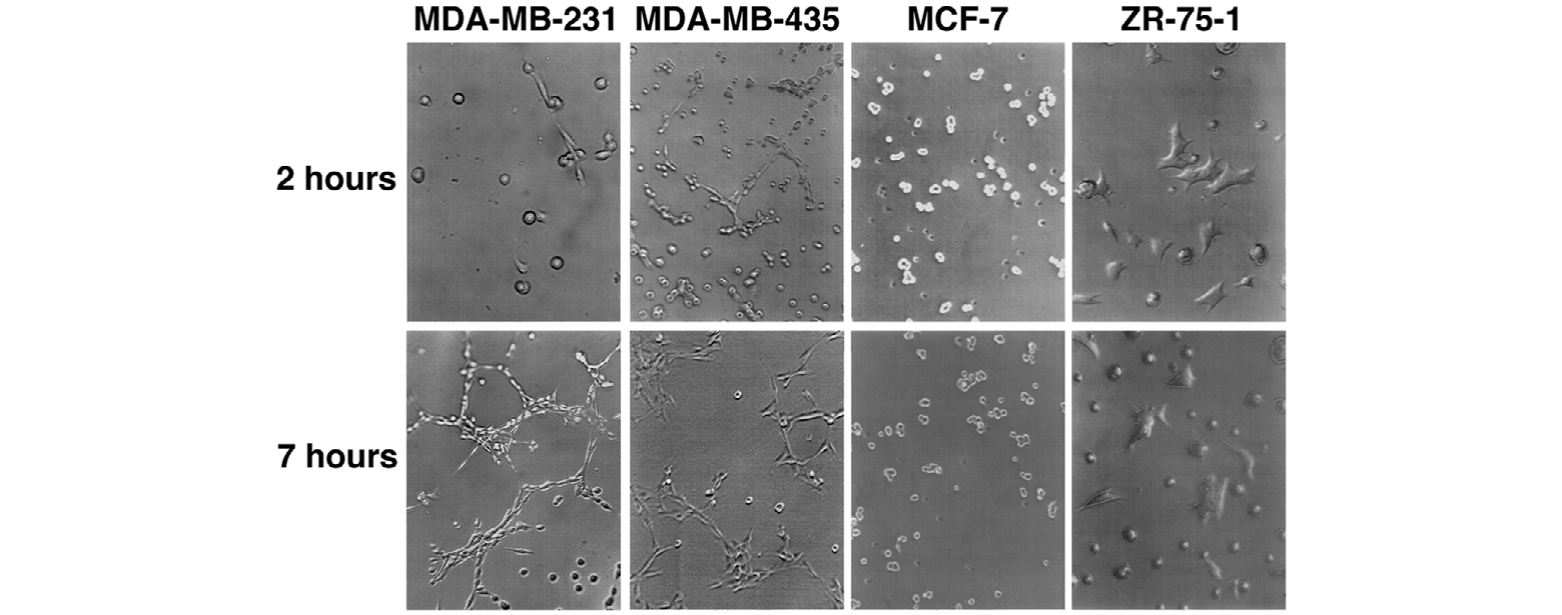
Kinetics of vascular channel formation by breast cancer cells. MDA-MB-231, MDA-MB-435, MCF-7, and ZR-75-1 cells were serum starved overnight, plated on matrigel, and images using phase contrast microscopy were taken at the times indicated. The MDA-MB-231 and MDA-MB-435 cells started to form vascular channels as early as 2 hours after plating on matrigel. Well defined patterned networks were observed by 7 hours. MCF-7 and ZR-75-1 cells failed to form patterned networks.

**Figure 2:**
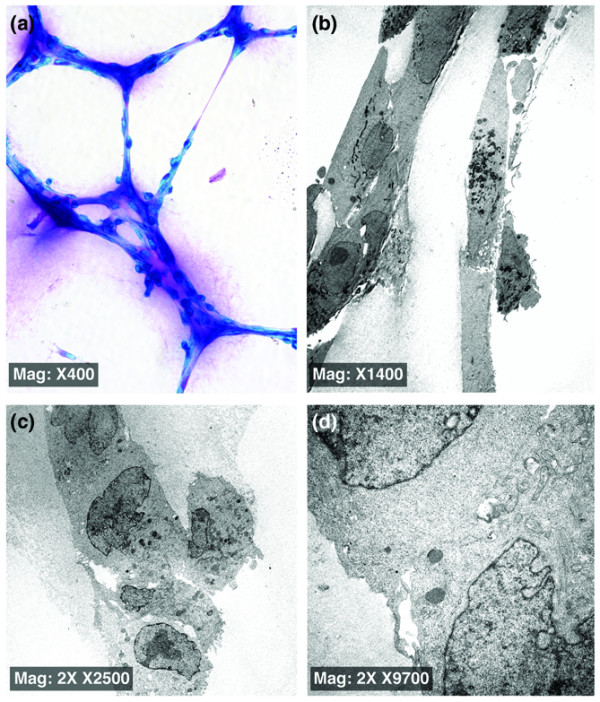
PAS staining and electron microscopic analysis of vascular channels formed by MDA-MB-231 cells. **(a) **PAS staining was performed on MDA-MB-231 cells plated on matrigel for 24 hours to identify the extracellular matrix secreted by cells. Pink staining refers to the glycogen and related mucopolysaccharides secreted by the cells to form the extracellular matrix-rich vascular channels. **(b) **Transmission electron microscopic analysis was performed on MDA-MB-231 cells plated on matrigel for 24 hours. A cluster of cells came into contact with each other to form vascular channels. There was cytoplasmic ruffling and protrusion at cell contact position. **(c) **There was evidence of stretching of cellular contents to form the tubular channel-like structure. **(d) **Absence of tight junctions between adjacent cells involved in vascular channel formation was observed. PAS, periodic acid-Schiff.

Both the highly invasive MDA-MB-231 cells [15], which over-express COX-2, and the moderately invasive MDA-MB-435 cells [21], which express moderate levels of COX-2, generated patterns that consisted of a translucent tubular network when plated on matrigel. These vascular tubular channels evolved dynamically and anastomosed over a 2 to 7 hour period (Figure 1). In contrast, the non-invasive MCF-7 and ZR-75-1 cells [22], which either lack or have very low expression of COX-2, did not form tubular networks on matrigel at 7 hours (Figure 1); channels were undetectable for up to 48 hours after plating (data not shown). In comparison with endothelial cell cords *in vitro *that typically demonstrate a uniform diameter on most matrices [23-26], the lumen diameter in these tubular networks varied widely. To highlight the matrix-associated vascular channels formed by the MDA-MB-231 cells, they were stained with periodic acid-Schiff, which identified the glycogen and related mucopolysaccharides secreted by the cells to form the extracellular-matrix-rich channels (Figure 2a). Fluid conductance within the channels was monitored using a fluorescence recovery after photobleaching (FRAP) assay on MDA-MB-231 cells stained with carboxy-fluorosuccinidylester (CFSE). Total recovery of fluorescence was observed within 10 to 12 seconds of photobleaching, suggesting active fluid conductive through the channels as opposed to passive diffusion process, which takes longer (data not shown).

### Electron microscopic analysis of MDA-MB-231 cells exhibiting vasculogenic mimicry

Ultrastructurally, the microvascular channels exhibited elongated tumor cells having oval euchromatic nuclei with prominent nucleoli (Figure 2b–d). The cytoplasm contained many mitochondria, well developed Golgi apparatus and vesicles, rough endoplasmic reticulum cisternae, and ribosomes. Extracellular basement membrane material was also noted. Numerous membrane bound granules of variable size and electron density were present. A cluster of cells contacted each other to form vascular channels, which were associated with cytoplasmic ruffling and protrusions at cell contact positions (Figure 2b). In Figure 2c we observed five cells stretching their cellular contents to form a tubular channel-like structure with no distinct plasma membrane between them. There was an absence of tight junctions between adjacent cells closely apposed to each other (Figure 2d). These morphologic features suggest that apposed cells become fused, which results in a channel-like structure lined by epithelial cells.

### Celecoxib inhibits vascular channel formation *in vitro*

**Figure 3:**
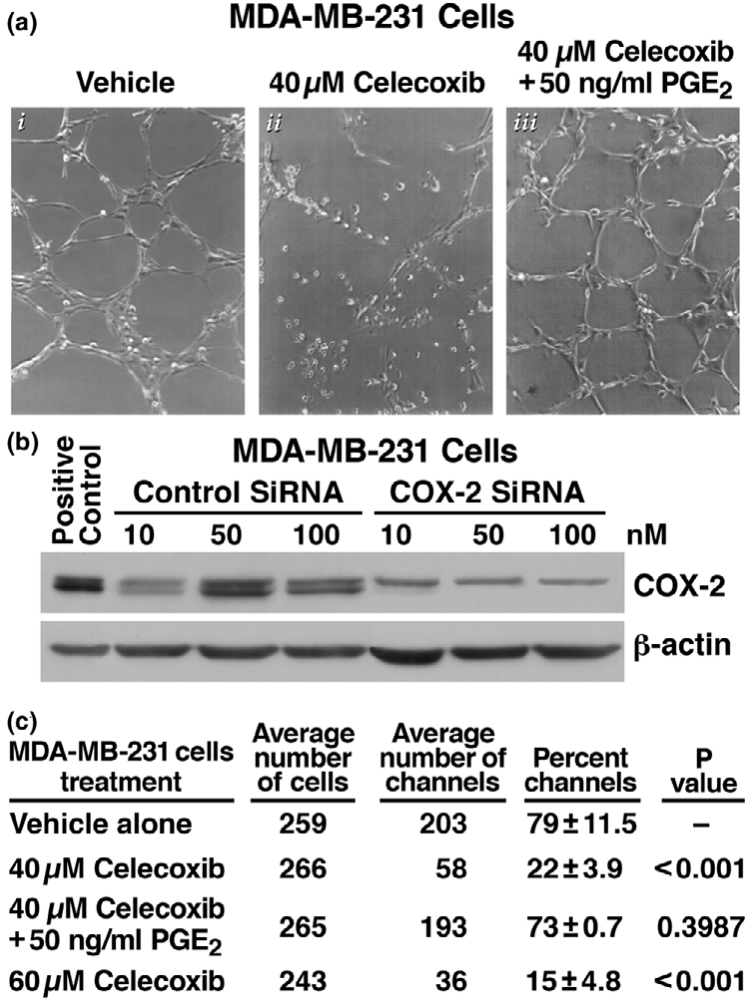
COX-2 inhibition by celecoxib or specific siRNA inhibits vascular channel formation. **(a) **Phase contrast images show vascular channel formation in growth factor reduced matrigel of MDA-MB-231, treated with vehicle or 40 mmol/l celecoxib. Images were captured 48 hours after plating using a phase contrast microscope. (part i) With vehicle treatment, MDA-MB-231 cells form well differentiated tubular structures. (part ii) With celecoxib treatment, differentiation into channels was significantly reduced in MDA-MB-231 cells. (part iii) Addition of 50 ng/ml PGE_2 _to MDA-MB-231 cells treated with 40 μM celecoxib could reverse the inhibitory effect of celecoxib. **(b) **COX-2 expression decreases in MDA-MB-231 cells with siRNA treatment. COX-2 protein expression was measured by Western blot. Treatment with a COX-2 siRNA for 48 hours significantly inhibited COX-2 expression at siRNA concentrations of 10, 50, and 100 nmol/l. Data shown are representative of three independent experiments. **(c) **Inhibition of vascular channel formation in MDA-MB-231 cells with celecoxib and COX-2-specific siRNA treatment. Quantitative analysis of vascular channel formation: the number of vascular channels was determined by counting the number of connected cells in five randomly selected fields, using 200 × magnification, and dividing that number by the total number of cells in the same field. Raw data from five standardized fields for each treatment from three separate experiments are shown. Treatment with 40 and 60 mmol/l celecoxib and treatment with a 50 nmol/l concentration of COX-2 siRNA for 48 hours caused significant decrease in the number of channels formed by MDA-MB-231 cells, and addition of 50 ng/ml of PGE_2 _was able to reverse the effect observed with treatment with 40 mmol/l celecoxib. *P *values represent significant difference between vehicle control and celecoxib treatment. COX, cyclo-oxygenase; PG, prostaglandin; siRNA, small interfering RNA.

Because COX-2 modulates several pathways during carcinogenesis including angiogenesis, we investigated the ability of COX-2 to regulate vascular channel formation. The MDA-MB-231 cells were chosen not only because they express high levels of COX-2, but also because they differentiate into tubular structures within 2 hours of plating on matrigel and form well defined vascular channels by 7 hours (Figure 1). We recently showed that treatment with celecoxib maximally affected growth of MDA-MB-231 cells at 48 hours after treatment [15], and so we tested the ability of celecoxib to affect vascular channel formation at 48 hours after treatment. We found that at 40 and 60 μmol/l doses, celecoxib treatment was able to reduce significantly the number of vascular channels formed by these cells as compared with cells treated with vehicle (*P *< 0.001; Figure 3 panels a [parts i and ii] and c), suggesting a role for COX-2 in channel formation. Similar reduction in channel formation was observed at 24 hours (data not shown). At a lower dose (20 μmol/l) of celecoxib, there was no effect on formation of vascular channels (data not shown). To determine whether celecoxib-induced inhibition of channel formation could be abrogated by adding exogenous PGE_2_, MDA-MB-231 cells were treated with 40 μmol/l celecoxib with or without varying dose of PGE_2 _(100 ng to 6.4 μg/ml). Addition of 50 ng/ml PGE_2 _completely restored channel formation in cells treated with 40 μmol/l celecoxib (Figure 3 panels a [part iii] and c), suggesting that the celecoxib-mediated inhibition was dependent on PGE_2_. Similar results were observed in other aggressive breast cancer cell lines (data not shown).

### Vascular channel formation decreased when MDA-MB-231 cells were treated with COX-2-specific siRNA

To verify our *in vitro *observation with celecoxib, we silenced the expression of COX-2 protein in MDA-MB-231 cells using siRNA technology. At all three concentrations (10, 50, and 100 nmol/l), the COX-2 siRNA oligos were able to significantly knock down COX-2 protein levels by 48 hours after transfection, as compared with control siRNA treated cells (Figure 3b). Because all three concentrations of the oligos were efficient in downregulating expression of COX-2, we selected 50 nmol/l (based on the manufacturer's recommendation) of the siRNA oligo treated cells for evaluation of channel formation. We observed a significant (*P *< 0.001) decrease in the number of channels formed by the COX-2 siRNA treated cells (50 channels) as compared with the control siRNA treated cells (175 channels; Figure 3c).

### Celecoxib treatment alters expression of genes associated with angiogenesis, proliferation, apoptosis, and cell cycle

**Figure 4:**
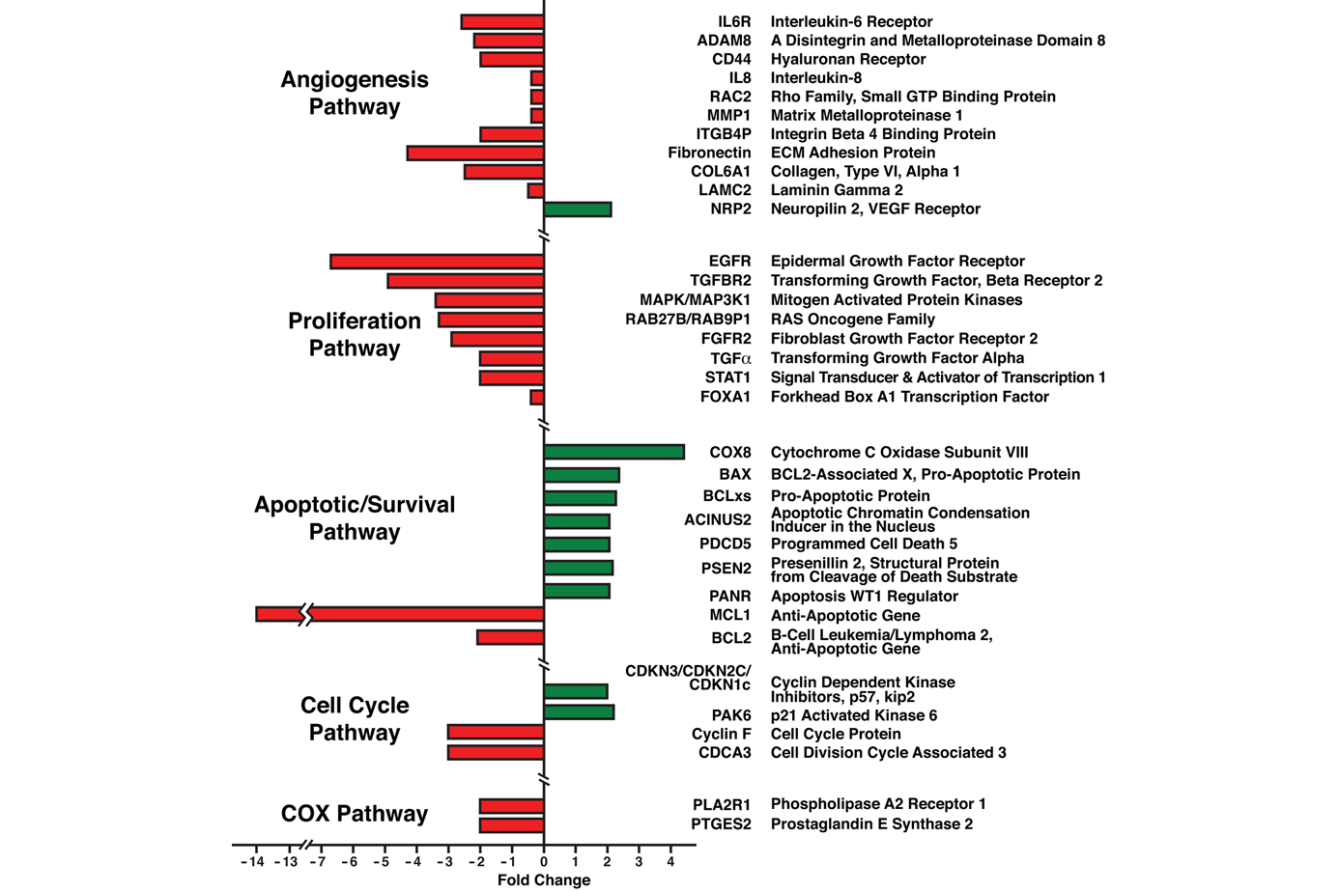
Treatment with celecoxib regulates gene expression in MDA-MB-231 cells. Differential gene expression was assessed by comparing MDA-MB-231 cells treated with or without 40 mmol/l celecoxib for 24 hours using the Affymetrix Human Genome 133A Gene Chip. Replicate microarray analyses were employed. A total of 44,760 genes were initially evaluated, and of these 1069 had a twofold or greater change. Selected results compare genes of interest associated with important cell pathways. Bars on the right represent an increase in gene expression and bars on the left indicate a decrease. COX, cyclo-oxygenase.

Because we established that inhibition of COX-2 decreases channel formation, we sought to elucidate the regulatory mechanism underlying the effect. To do so, differential gene expression was evaluated by comparing cells plated on three-dimensional matrigel cultures that were either treated with vehicle or 40 μmol/l celecoxib for 24 hours using expression profiling with Affymetrix Human Genome 133A (Affymetrix Inc., Santa Clara, CA, USA). As illustrated in Figure 4, numerous gene expression patterns were altered in association with treatment. A treatment/vehicle signal ratio (fold change) of = 2 or = 0.5 was considered a significant induction or repression, consistent with previous reports of microarray gene expression analysis [27,28]. Briefly summarized, of the 1069 known genes that were differentially expressed in these two treatment conditions, approximately 35 have been previously associated with the angiogenic, proliferative, apoptotic/survival, cell cycle, and COX pathways (Figure 4). This is consistent with the view that COX-2 inhibitors have multiple mechanisms of action as cancer chemopreventive agents.

A series of genes that could generate relevant biologic molecules to form vascular channels were observed to be downregulated with celecoxib treatment, including fibronectin, collagen, and laminin. Similarly, in the angiogenic pathway, celecoxib treatment caused downregulation of IL-6 receptor, the ADAM8 (a disintegrin and metalloproteinase domain 8) gene, CD44 and integrin-β binding proteins, and upregulation of neuropilin 2 (a VEGF receptor). In addition, celecoxib affected the proliferation pathway by causing decreased expression of epidermal growth factor (EGF) receptor, FGF receptor 2, transforming growth factor-β receptor 2, mitogen-activated protein kinase, Ras oncogenes, transforming growth factor-α, and STAT1 (signal transducer and activator of transcription 1). Activation of the apoptotic/survival machinery was also observed, whereby celecoxib treatment induced expression of genes encoding such products as cytochrome C oxidase subunit VIII, BAX, BCLxs, presenillin 2 (structural protein from cleavage of death substrate), apoptotic chromatin condensation inducer, and PDCD5 (programmed cell death 5). Concomitantly, antiapoptotic genes such as MCL1 and BCL2 were downregulated. Consistent with the expression data, we previously showed a significant increase in BAX protein and decrease in BCL2 protein on celecoxib treatment [15,29]. Celecoxib also affected the cell cycle pathway by causing an upregulation of cyclin-dependent kinase inhibitors p57, p21 and kip2, and downregulation of cyclin F and cell division cycle associated 3 proteins. Finally, in the COX pathway, celecoxib treatment caused downregulation of phospholipase A2 receptor 1 and prostaglandin E synthase 2, which is consistent with our previous report in which PGE_2 _protein was shown to be significantly downregulated after celecoxib treatment [15,29].

In summary, the expression analysis allowed us to begin to elucidate the mechanisms by which celecoxib may play a role in reducing VM and angiogenesis.

### Celecoxib treatment caused decreased expression of angiogenic proteins

**Figure 5:**
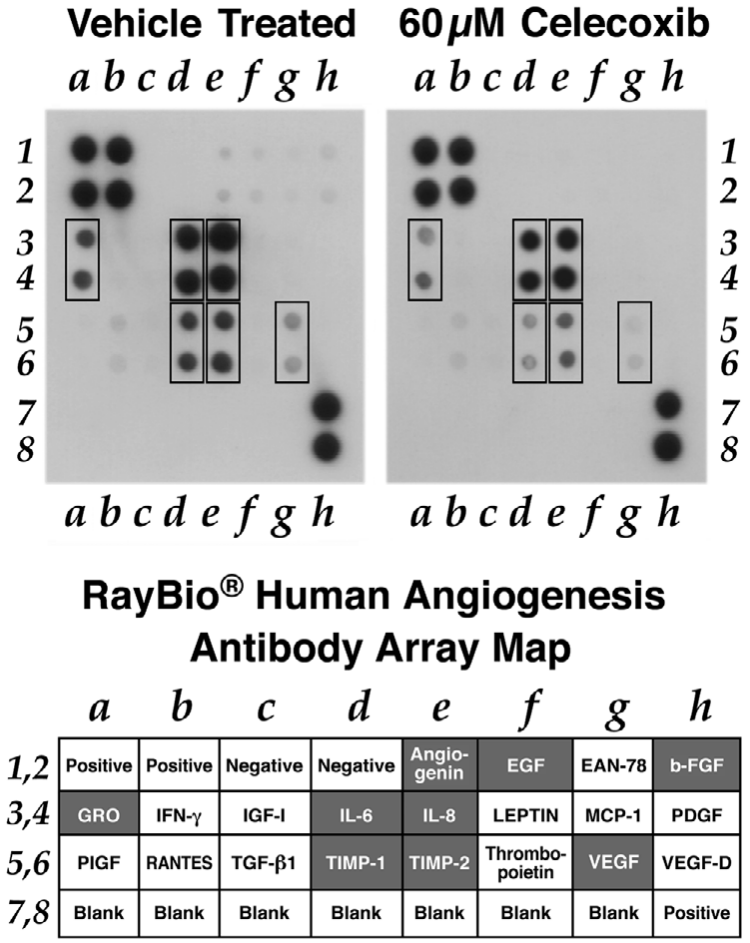
Cell supernatant analysis of angiogenic proteins revealed decreased levels with celecoxib treatment. The cell culture supernatants from MDA-MB-231 cells treated with vehicle and 40 mmol/l celecoxib for 24 hours were tested on a human angiogenesis protein array. The cell culture supernatants of cells treated with vehicle had higher amounts of GRO, IL-6, IL-8, TIMP1, TIMP2, and VEGF based on gray levels or brightness values as compared with cells treated with celecoxib. Other proteins affected by treatment were EGF, bFGF, and angiogenin. The array template is shown in the lower panel, with dark gray indicating high expression. bFGF, basic fibroblast growth factor; EGF, epidermal growth factor; IFN, interferon; IGF, insulin-like growth factor; IL, interleukin; MCP, monocyte chemoattractant protein; PDGF, platelet-derived growth factor; PG, prostaglandin; RANTES, regulated on activation, normal T cell expressed and secreted; TIMP, tissue inhibitor of matrix metalloproteinase; VEGF, vascular endothelial growth factor.

The cell culture supernatant from MDA-MB-231 cells treated with vehicle or celecoxib (40 μmol/l) for 24 hours was tested on a human angiogenesis array. Several angiogenic proteins were downregulated by celecoxib on membrane arrays containing 20 different angiogenic proteins (Figure 5). The cell culture supernatants of vehicle treated cells had greater amounts of growth related protein (GRO), IL-6, IL-8, tissue inhibitor of matrix metalloproteinase (TIMP)1 and TIMP2, and VEGF, based on gray levels or brightness values as compared with cells treated with celecoxib. Other proteins affected were EGF, bFGF, and angiogenin, which were already low in the supernatant of cells, treated with vehicle, but were not detected in supernatants of cells treated with celecoxib. The presence of these proteins was not due to the fetal bovine serum in the DMEM medium, because incubation with the medium alone did not result in reactivity with any of the antibody spots (data not shown).

### Celecoxib treatment *in vivo *inhibited vasculogenic mimicry

**Figure 6:**
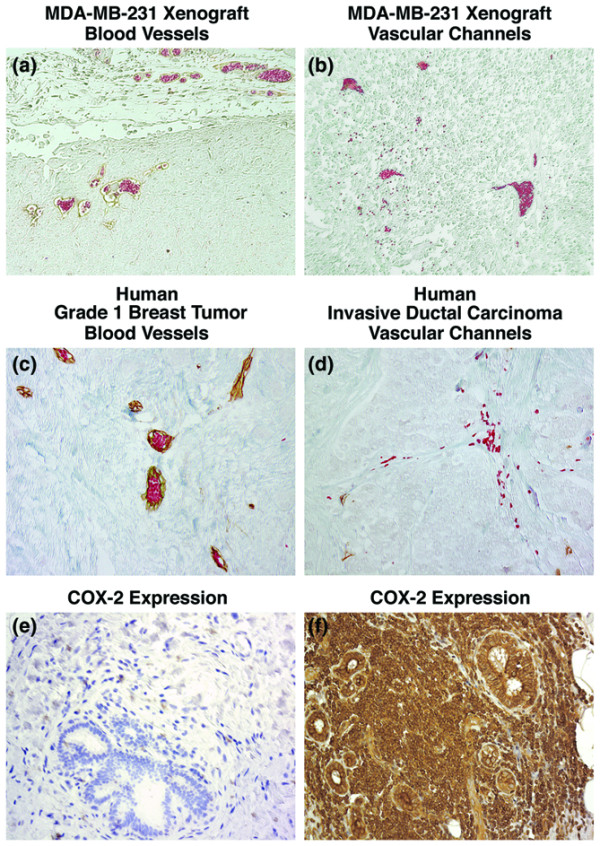
*In vivo *inhibition of VM with treatment and presence of VM in breast cancer specimens. **(a) **Vascularity of tumor xenografts in mice was evaluated by factor VIII related antigen staining (brown) for endothelial cells, and Biebrich Scarlet staining (red) for RBCs. **(b) **Absence of endothelial cells lining the pools of RBCs (vascular channels) was shown by no staining for factor VIII related antigen and positive staining for RBC by Biebrich Scarlet. **C) **Histological features of the primary human grade 1 breast tumor specimen showing blood vessels positive for CD34 staining (brown) for endothelial cells, and Biebrich Scarlet staining (red) for RBCs. No vascular channels were detected. **(d) **RBC pooling without the lining of endothelium (vascular channels) in the high-grade invasive ductal breast carcinoma specimen. **(e, f) **COX-2 staining pattern in grade 1 primary human breast tumor specimen (panel e) and in high-grade invasive ductal carcinoma specimen (penal f). Magnifications are as follows: panels a and b, 100 ×; panels c and d, 200 ×; and panels E and F, 100 × magnification). COX, cyclo-oxygenase; RBC, red blood cell; VM, vasculogenic mimicry.

Presence of blood vessels lined by endothelial cells was evaluated by factor VIII related antigen staining and Biebrich Scarlet staining for RBCs (Figure 6a). Blood vessels were detected predominantly in the capsular and peripheral regions of the MDA-MB-231 transplanted tumors excised from athymic nude mice. In contrast to blood vessels, which were lined by endothelial cells, vascular channels were identified as vessels in the tumors containing erythrocytes (detected by Biebrich Scarlet staining) but that lacked endothelial cell lining (determined by the lack of factor VIII staining; Figure 6b). Previously, we showed that blood vessels lined by endothelial cells (vascularization) were significantly decreased in tumor implants dissected from mice treated with celecoxib [15]. Similarly, tumor weight was significantly (*P *= 0.01) lower in mice treated with celecoxib than in mice treated with vehicle (0.6 ± 0.1 g versus 1.9 ± 0.1 g, respectively; *n *= 5 mice/group) [15]. In the present study we extend our observation to vascular channel formation in mice treated with celecoxib. Tumors excised from mice treated with celecoxib were devoid of vascular channels (data not shown). Furthermore, the presence of vascular channels was only detected in the central hypoxic sections of the tumors from mice treated with vehicle (Figure 6b), confirming that the vascular channels may be used to obtain nutrients in less vascularized tumor regions.

### Vasculogenic mimicry was detected in high-grade human breast cancer tissues

We identified VM in three out of ten cases of high-grade (grade 3) tumors examined. Vascular channels were detected by staining tumor specimens with Biebrich Scarlet for RBCs and anti-CD34 antibody for endothelial cells. Vascular channels were not lined by endothelial cells as evidenced by the lack of CD34 (brown) staining around the pool of RBCs (Figure 6d). In comparison, typical blood vessels exhibit RBCs in spaces lined by endothelial cells (brown stain; Figure 6c). VM was not observed in any of the low-grade breast cancer specimens examined (*n *= 10 cases). Human high-grade invasive tumor specimens that expressed high levels of COX-2 proteins (Figure 6f) had detectable vascular channels, whereas low-grade tumors with no or low COX-2 expression (Figure 6e) had little evidence of VM. Representative COX-2 staining patterns for a high-grade metastatic ductal carcinoma and a low-grade (grade 1) non-invasive tumor are shown (Figure 6e,f).

## Discussion

Vascular channels can be formed by epithelial tumor cells and are detected in several high-grade invasive tumor specimens, indicating that endothelial cell mediated angiogenesis is not the only mechanism providing nourishment to tumors and metastatic lesions [2-4]. Our study addresses the intriguing cellular and molecular mechanisms underlying the role of COX-2 in vascular channel formation in human breast cancer cells. The data reveal that COX-2 plays a vital role in vascular channel formation by breast cancer cells *in vitro *and *in vivo*. First, we found that only the highly invasive breast cancer cells with high levels of COX-2 form patterned vascular channels, and these channels are different from endothelium-derived angiogenic vessels. Second, inhibiting COX-2 reduced channel formation, and addition of exogenous PGE_2 _restored channel formation. Finally, we identified vascular channels in necrotic areas of primary human high-grade invasive breast cancer specimens that express high levels of COX-2, suggesting that these channels may serve as an alternative means of generating microcirculation in hypoxic regions of the tumor and thus facilitate metastasis.

Highly invasive MDA-MB-231 and less invasive MDA-MB-435 cells form patterned matrix-associated vascular channels *in vitro*. In contrast, poorly invasive ZR-75-1 and MCF-7 cell lines are not able to generate patterned vascular channels (Figure 1). A number of markers expressed by the MDA-MB-231 cells, including thrombin receptor, TIE-2, CD31, VEGF, and bc-48, have been shown to be associated with endothelial cells [30-34]. The presence of these typical endothelial proteins expressed in the MDA-MB-231 cells may increase invasive and metastatic activity, and help to elucidate the molecular mechanisms that underlie the channel-forming ability of these highly aggressive tumor cells. The ability of the highly invasive COX-2^high ^but not the poorly invasive COX-2^low ^breast cancer cell lines to generate patterned vascular channels *in vitro *correlates well with the presence of vascular channels only in the high-grade, COX-2^high ^invasive human ductal adenocarcinoma specimens (Figure 6c–f).

We therefore hypothesized that COX-2 may play a regulatory role in vascular channel formation in breast cancer. The ability of MDA-MB-231 cells to differentiate into channels was significantly reduced when they were treated with increasing doses of celecoxib (40 to 60 μmol/l; Figure 3a,c). These results were verified by downregulating COX-2 protein expression using the siRNA approach (Figure 3b). The effect of celecoxib to inhibit vascular channels was dependent on PGE_2_, because adding exogenous PGE_2 _to cultures restored the ability of cells to form channels. Differential gene expression analysis suggests that celecoxib treatment, impinging on VM, has impacts on multiple pathways including angiogenesis. Decreased expression of angiogenic proteins such as IL-6R, ADAM family genes, CD44, IL-8, Rho family genes, integrin-β binding proteins, MMPs, and laminin was observed with celecoxib treatment in MDA-MB-231 cells. In melanoma cells, it has been reported that in the VM signaling cascade there is activation of phosphatidylinositol-3 kinase, which in turn promotes the activity of MMPs and cleavage of laminin 5γ 2 chain, releasing signals into the tumor microenvironment to execute VM [5]. In our study celecoxib was able to neutralize the inductive potential of the tumor cell microenvironment by decreasing levels of MMPs, TIMP1, TIMP2, and laminin (Figures 4 and 5). Consistent with a significant decrease in PGE_2 _secretion by MDA-MB-231 cells after celecoxib treatment [15], we observed a twofold decrease in prostaglandin E synthase 2 (Figure 4), which is the enzyme involved in the synthesis of PGE_2_. This is particularly relevant because COX-2-dependent PGE_2 _represents a likely candidate for the angiogenic response observed in several tumors, including mammary tumors [29,35-38].

Vascular mimicry and COX-2 are both associated with angiogenesis [39], and gene expression data implicated genes in the angiogenic pathway, and so we evaluated the angiogenic proteins affected by celecoxib during vascular channel formation using an angiogenesis protein array. The major angiogenic proteins downregulated by celecoxib treatment were VEGF, GRO, IL-6, IL-8, TIMP1, and TIMP2 (Figure 5). The array data confirm our previous data on dose-dependent inhibition of VEGF in MDA-MB-231 cells after celecoxib treatment [15] and our gene expression data. Of interest is the increase in the neuropillin 2 VEGF receptor gene expression (Figure 4) but a decrease in VEGF levels (Figure 5), suggesting a novel autocrine feedback mechanism during VM. Furthermore, there were decreases in EGF, transforming growth factor-α, and bFGF levels upon celecoxib treatment, both at gene and protein levels (Figures 4 and 5). It is well documented that tumor cells synthesize and respond to growth factors such as EGF, FGF, and PDGF [40], and that NSAIDs negatively regulate the EGF/PDGF pathway with evidence of crosstalk between COX-2 and EGFR [41-43].

Because celecoxib treatment also regulates genes in the apoptosis and cell cycle pathways (Figure 4), it may be argued that the negative effect on vascular channel formation may be in part an indirect effect of apoptosis and/or cell cycle arrest. However, it is important to note that the gene expression analysis was performed only on live, adherent cells, and genes involved in vascular channel formation including fibronectin, collagen, and laminin were downregulated with celecoxib treatment. Thus, we suggest that celecoxib may have an independent effect on vascular channel formation.

To confirm the *in vitro *data, the effects of celecoxib on channel formation and angiogenesis were evaluated in an *in vivo *xenograft model using MDA-MB-231 cells. With celecoxib treatment *in vivo*, we were unable to detect any channel formation. In contrast, vehicle-treated tumors exhibited substantial vascular channel formation specifically in necrotic areas of the tumor (Figure 6b). Previous studies have reported similar effects of COX-2 inhibitors on angiogenesis using the murine mammary tumor cell line C3L5 [12]; however, there have been no reports thus far on the effect of COX-2 inhibitor on vascular channel formation by breast cancer cells. Additional studies are needed to elucidate fully the complex events involved in COX-2-mediated vascularization and channel formation in primary human tumors.

Of relevance is the occurrence of VM in three out of ten primary human high-grade invasive tumor specimens, with no evidence of VM in low-grade tumors (*n *= 10; Figure 6c,d). Thus far, there has only been one report of VM in primary human IBC specimens in which the authors correlated VM with high expression of Flt-1 and TIE-2 and absence of CD31 and thrombin [7]. Our studies suggest a correlation between channel formation and high COX-2 expression. Although we recognize that not all breast cancer specimens that were COX-2-positive exhibited VM, our evaluation implies that specimens lacking channel formation were always negative for COX-2. Inhibiton of COX-2 may therefore be considered part of a treatment regimen for patients with high-grade invasive ductal carcinomas. Interestingly, one of the three specimens that were positive for VM was obtained from a patients with IBC.

## Conclusion

Our data, for the first time, implicate COX-2 as a regulator of vascular channel formation in human breast cancer cells. The data demonstrate a mechanistic role for COX-2 in vascular channel formation, with significant inhibition of channel formation when COX-2 is specifically inhibited. Gene expression and protein array data implicate several factors that help to explain the molecular basis underlying the unique architecture of vascular channels formed by aggressive breast cancer cells and regulated by COX-2.

## Abbreviations

bFGF = basic fibroblast growth factor; COX = cyclo-oxygenase; DMEM = Dulbecco's modified Eagle's medium; EGF = epidermal growth factor; ERK = extracellular signal-regulated kinase; GRO = growth related protein; IBC = inflammatory breast cancer; IL = interleukin; MMP = matrix metalloproteinase; PG = prostaglandin; RBC = red blood cell; siRNA = small interfering RNA; TIMP = tissue inhibitor of matrix metalloproteinase; VEGF = vascular endothelial growth factor; VM = vasculogenic mimicry.

## Competing interests

The authors declare that they have no competing interests.

## Authors' contributions

Dr Gargi D Basu is a senior postdoctoral fellow, and carried out the experiments and wrote the manuscript. Winnie S Liang is the technician in Dr Dietrich A Stephan's laboratory in TGen, and conducted the gene expression experiments. Drs Lee T Wegener, Christopher R Conley and Barbara A Pockaj are Mayo Clinic physicians and were critical to the procurement and analysis of human pathologic samples, and Dr Pinku Mukherjee is the principal investigator of the laboratory in which the research was performed.

## References

[B1] Folkman J, Klagsbrun M (1987). Angiogenic factors. Science.

[B2] Pezzella F, Pastorino U, Tagliabue E, Andreola S, Sozzi G, Gasparini G, Menard S, Gatter KC, Harris AL, Fox S (1997). Non-small-cell lung carcinoma tumor growth without morphological evidence of neo-angiogenesis. Am J Pathol.

[B3] Maniotis AJ, Folberg R, Hess A, Seftor EA, Gardner LM, Pe'er J, Trent JM, Meltzer PS, Hendrix MJ (1999). Vascular channel formation by human melanoma cells *in vivo* and *in vitro*: vasculogenic mimicry. Am J Pathol.

[B4] Shirakawa K, Shibuya M, Heike Y, Takashima S, Watanabe I, Konishi F, Kasumi F, Goldman CK, Thomas KA, Bett A (2002). Tumor-infiltrating endothelial cells and endothelial precursor cells in inflammatory breast cancer. Int J Cancer.

[B5] Hendrix MJ, Seftor EA, Hess AR, Seftor RE (2003). Vasculogenic mimicry and tumour-cell plasticity: lessons from melanoma. Nat Rev Cancer.

[B6] Shirakawa K, Kobayashi H, Heike Y, Kawamoto S, Brechbiel MW, Kasumi F, Iwanaga T, Konishi F, Terada M, Wakasugi H (2002). Hemodynamics in vasculogenic mimicry and angiogenesis of inflammatory breast cancer xenograft. Cancer Res.

[B7] Shirakawa K, Wakasugi H, Heike Y, Watanabe I, Yamada S, Saito K, Konishi F (2002). Vasculogenic mimicry and pseudo-comedo formation in breast cancer. Int J Cancer.

[B8] Hendrix MJ, Seftor EA, Meltzer PS, Gardner LM, Hess AR, Kirschmann DA, Schatteman GC, Seftor RE (2001). Expression and functional significance of VE-cadherin in aggressive human melanoma cells: role in vasculogenic mimicry. Proc Natl Acad Sci USA.

[B9] Seftor RE, Seftor EA, Koshikawa N, Meltzer PS, Gardner LM, Bilban M, Stetler-Stevenson WG, Quaranta V, Hendrix MJ (2001). Cooperative interactions of laminin 5 gamma2 chain, matrix metalloproteinase-2, and membrane type-1-matrix/metalloproteinase are required for mimicry of embryonic vasculogenesis by aggressive melanoma. Cancer Res.

[B10] Shirakawa K, Tsuda H, Heike Y, Kato K, Asada R, Inomata M, Sasaki H, Kasumi F, Yoshimoto M, Iwanaga T (2001). Absence of endothelial cells, central necrosis, and fibrosis are associated with aggressive inflammatory breast cancer. Cancer Res.

[B11] van der Schaft DW, Seftor RE, Seftor EA, Hess AR, Gruman LM, Kirschmann DA, Yokoyama Y, Griffioen AW, Hendrix MJ (2004). Effects of angiogenesis inhibitors on vascular network formation by human endothelial and melanoma cells. J Natl Cancer Inst.

[B12] Rozic JG, Chakraborty C, Lala PK (2001). Cyclooxygenase inhibitors retard murine mammary tumor progression by reducing tumor cell migration, invasiveness and angiogenesis. Int J Cancer.

[B13] Tsujii M, Kawano S, Tsuji S, Sawaoka H, Hori M, DuBois RN (1998). Cyclooxygenase regulates angiogenesis induced by colon cancer cells. Cell.

[B14] Jones MK, Wang H, Peskar BM, Levin E, Itani RM, Sarfeh IJ, Tarnawski AS (1999). Inhibition of angiogenesis by nonsteroidal anti-inflammatory drugs: insight into mechanisms and implications for cancer growth and ulcer healing. Nat Med.

[B15] Basu GD, Pathangey LB, Tinder TL, Gendler SJ, Mukherjee P (2005). Mechanisms underlying the growth inhibitory effects of COX-2 inhibitor in human breast cancer cells. Breast Cancer Res.

[B16] Williams CS, Watson AJ, Sheng H, Helou R, Shao J, DuBois RN (2000). Celecoxib prevents tumor growth in vivo without toxicity to normal gut: lack of correlation between *in vitro* and *in vivo* models. Cancer Res.

[B17] Hsu AL, Ching TT, Wang DS, Song X, Rangnekar VM, Chen CS (2000). The cyclooxygenase-2 inhibitor celecoxib induces apoptosis by blocking Akt activation in human prostate cancer cells independently of Bcl-2. J Biol Chem.

[B18] Lai GH, Zhang Z, Sirica AE (2003). Celecoxib acts in a cyclooxygenase-2-independent manner and in synergy with emodin to suppress rat cholangiocarcinoma growth *in vitro* through a mechanism involving enhanced Akt inactivation and increased activation of caspases-9 and -3. Mol Cancer Ther.

[B19] DeRisi J, Penland L, Brown PO, Bittner ML, Meltzer PS, Ray M, Chen Y, Su YA, Trent JM (1996). Use of a cDNA microarray to analyse gene expression patterns in human cancer. Nat Genet.

[B20] Khan J, Simon R, Bittner M, Chen Y, Leighton SB, Pohida T, Smith PD, Jiang Y, Gooden GC, Trent JM, Meltzer PS (1998). Gene expression profiling of alveolar rhabdomyosarcoma with cDNA microarrays. Cancer Res.

[B21] Gilhooly EM, Rose DP (1999). The association between a mutated ras gene and cyclooxygenase-2 expression in human breast cancer cell lines. Int J Oncol.

[B22] Half E, Tang XM, Gwyn K, Sahin A, Wathen K, Sinicrope FA (2002). Cyclooxygenase-2 expression in human breast cancers and adjacent ductal carcinoma *in situ*. Cancer Res.

[B23] Folkman J, Haudenschild C (1980). Angiogenesis in vitro. Nature.

[B24] Ingber DE, Folkman J (1989). Mechanochemical switching between growth and differentiation during fibroblast growth factor-stimulated angiogenesis *in vitro*: role of extracellular matrix. J Cell Biol.

[B25] Nicosia RF, Ottinetti A (1990). Modulation of microvascular growth and morphogenesis by reconstituted basement membrane gel in three-dimensional cultures of rat aorta: a comparative study of angiogenesis in matrigel, collagen, fibrin, and plasma clot. In Vitro Cell Dev Biol.

[B26] Vernon RB, Sage EH (1995). Between molecules and morphology. Extracellular matrix and creation of vascular form. Am J Pathol.

[B27] Dong Y, Zhang H, Hawthorn L, Ganther HE, Ip C (2003). Delineation of the molecular basis for selenium-induced growth arrest in human prostate cancer cells by oligonucleotide array. Cancer Res.

[B28] Chen CR, Kang Y, Massague J (2001). Defective repression of c-myc in breast cancer cells: A loss at the core of the transforming growth factor beta growth arrest program. Proc Natl Acad Sci USA.

[B29] Basu GD, Pathangey LB, Tinder TL, Lagioia M, Gendler SJ, Mukherjee P (2004). COX-2 inhibitor induces apoptosis in breast cancer cells in an *in vivo* model of spontaneous metastatic breast cancer. Mol Cancer Res.

[B30] Henrikson KP, Salazar SL, Fenton JW 2nd, Pentecost BT (1999). Role of thrombin receptor in breast cancer invasiveness. Br J Cancer.

[B31] Cramer EM, Berger G, Berndt MC (1994). Platelet alpha-granule and plasma membrane share two new components: CD9 and PECAM-1. Blood.

[B32] Jones N, Master Z, Jones J, Bouchard D, Gunji Y, Sasaki H, Daly R, Alitalo K, Dumont DJ (1999). Identification of Tek/Tie2 binding partners. Binding to a multifunctional docking site mediates cell survival and migration. J Biol Chem.

[B33] Tsopanoglou NE, Maragoudakis ME (1999). On the mechanism of thrombin-induced angiogenesis. Potentiation of vascular endothelial growth factor activity on endothelial cells by up-regulation of its receptors. J Biol Chem.

[B34] Takahama M, Tsutsumi M, Tsujiuchi T, Nezu K, Kushibe K, Taniguchi S, Kotake Y, Konishi Y (1999). Enhanced expression of Tie2, its ligand angiopoietin-1, vascular endothelial growth factor, and CD31 in human non-small cell lung carcinomas. Clin Cancer Res.

[B35] Chang SH, Liu CH, Conway R, Han DK, Nithipatikom K, Trifan OC, Lane TF, Hla T (2004). Role of prostaglandin E2-dependent angiogenic switch in cyclooxygenase 2-induced breast cancer progression. Proc Natl Acad Sci USA.

[B36] Ben-Av P, Crofford LJ, Wilder RL, Hla T (1995). Induction of vascular endothelial growth factor expression in synovial fibroblasts by prostaglandin E and interleukin-1: a potential mechanism for inflammatory angiogenesis. FEBS Lett.

[B37] Seno H, Oshima M, Ishikawa TO, Oshima H, Takaku K, Chiba T, Narumiya S, Taketo MM (2002). Cyclooxygenase 2- and prostaglandin E(2) receptor EP(2)-dependent angiogenesis in Apc(Delta716) mouse intestinal polyps. Cancer Res.

[B38] Chu J, Lloyd FL, Trifan OC, Knapp B, Rizzo MT (2003). Potential Involvement of the Cyclooxygenase-2 pathway in the regulation of tumor-associated angiogenesis and growth in pancreatic cancer. Mol Cancer Ther.

[B39] Shirakawa K, Kobayashi H, Sobajima J, Hashimoto D, Shimizu A, Wakasugi H (2003). Inflammatory breast cancer: vasculogenic mimicry and its hemodynamics of an inflammatory breast cancer xenograft model. Breast Cancer Res.

[B40] Aaronson SA (1991). Growth factors and cancer. Science.

[B41] Chu EC, Chai J, Tarnawski AS (2004). NSAIDs activate PTEN and other phosphatases in human colon cancer cells: novel mechanism for chemopreventive action of NSAIDs. Biochem Biophys Res Commun.

[B42] Dannenberg AJ, Lippman SM, Mann JR, Subbaramaiah K, DuBois RN (2005). Cyclooxygenase-2 and epidermal growth factor receptor: pharmacologic targets for chemoprevention. J Clin Oncol.

[B43] Dannenberg AJ, Subbaramaiah K (2003). Targeting cyclooxygenase-2 in human neoplasia: rationale and promise. Cancer Cell.

